# Aluminum Alters the Histology and Pectin Cell Wall Composition of Barley Roots

**DOI:** 10.3390/ijms20123039

**Published:** 2019-06-21

**Authors:** Joanna Jaskowiak, Jolanta Kwasniewska, Anna Milewska-Hendel, Ewa Urszula Kurczynska, Miriam Szurman-Zubrzycka, Iwona Szarejko

**Affiliations:** 1 Department of Plant Anatomy and Cytology, University of Silesia in Katowice, Jagiellonska 28, 40-032 Katowice, Poland; joanna_jaskowiak@wp.eu; 2 Department of Cell Biology, University of Silesia in Katowice, Jagiellonska 28, 40-032 Katowice, Poland; anna.milewska@us.edu.pl; 3 Department of Genetics, University of Silesia in Katowice, Jagiellonska 28, 40-032 Katowice, Poland; miriam.szurman@us.edu.pl (M.S.-Z.); iwona.szarejko@us.edu.pl (I.S.)

**Keywords:** aluminum, barley, cell wall, pectins, root

## Abstract

Aluminum (Al) is one of the most important crust elements causing reduced plant production in acidic soils. Barley (*Hordeum vulgare* L.) is considered to be one of the crops that is most sensitive to Al, and the root cell wall is the primary target of Al toxicity. In this study, we evaluate the possible involvement of specific pectic epitopes in the cells of barley roots in response to aluminum exposure. We targeted four different pectic epitopes recognized by LM5, LM6, LM19, and LM20 antibodies using an immunocytochemical approach. Since Al becomes available and toxic to plants in acidic soils, we performed our analyses on barley roots that had been grown in acidic conditions (pH 4.0) with and without Al and in control conditions (pH 6.0). Differences connected with the presence and distribution of the pectic epitopes between the control and Al-treated roots were observed. In the Al-treated roots, pectins with galactan sidechains were detected with a visually lower fluorescence intensity than in the control roots while pectins with arabinan sidechains were abundantly present. Furthermore, esterified homogalacturonans (HGs) were present with a visually higher fluorescence intensity compared to the control, while methyl-esterified HGs were present in a similar amount. Based on the presented results, it was concluded that methyl-esterified HG can be a marker for newly arising cell walls. Additionally, histological changes were detected in the roots grown under Al exposure. Among them, an increase in root diameter, shortening of root cap, and increase in the size of rhizodermal cells and divisions of exodermal and cortex cells were observed. The presented data extend upon the knowledge on the chemical composition of the cell wall of barley root cells under stress conditions. The response of cells to Al can be expressed by the specific distribution of pectins in the cell wall and, thus, enables the knowledge on Al toxicity to be extended by explaining the mechanism by which Al inhibits root elongation.

## 1. Introduction

Aluminum (Al) is the third most abundant element in the Earth’s crust and the most common metal. At neutral pH, it is present in soils as insoluble aluminosilicates and oxides which are harmless to living organisms [1]. However, in pH below 5.5, Al becomes soluble and available to plants in the form of phytotoxic Al^3+^ ions [2]. Al-induced changes in the root system reduce nutrient uptake, which results in a nutritional deficiency that leads to a reduction of overall plant growth and yield. It is estimated that about 50% of the world’s arable lands are acidic, predominantly in South America, Central Africa, and Southwest Asia, but also in eastern North America and throughout Europe [3]. Additionally, industrial pollution and the application of ammonium-containing fertilizers promote soil acidification [4]. In developing countries, where the use of amendments in order to raise the soil pH is limited by economic constraints, the problem of soil acidity is most prevalent, but Al toxicity is considered to be one of the most important limiting factors in agricultural production worldwide [5].

The first symptom of aluminum toxicity is the inhibition of root growth, which is correlated with a decrease in cell divisions and cell elongation in the root tip [6]. Research on *Arabidopsis* has indicated that the inhibition of cell divisions and cell cycle arrest in root meristems is a result of the activation of the DDR (DNA damage response) pathway which is induced by Al ions [7]. Cell elongation, the other component of reduced root growth under Al exposure, is connected with modifications in cell wall composition [8]. The cell wall is the first barrier that Al meets during exposure and is the primary target of Al toxicity [9]. Plants differ in their tolerance to Al and barley (*Hordeum vulgare* L.)—the fourth-ranked cereal species with regard to world productivity and cultivation area—is one of the most sensitive crops [10]. Our previous studies on the effects of Al in barley roots were focused on DNA and its integrity in response to Al treatment. Both cytotoxic and genotoxic effects of Al were observed in barley as a decrease in the mitotic activity of roots, along with the formation of damaged micronuclei and nuclei resulting from DNA breaks, as well as the inhibition of DNA replication and changes in the cell cycle profile [11]. In those studies, we observed ‘a stubby and brittle’ phenotype of the barley roots exposed to Al, which has been described by many authors as a symptom of an effect of Al on the cell wall [12]. As an integral component of a plant cell, the wall changes in response to internal and external factors and stresses, including Al [8]. The major role of the apoplast in Al perception is widely accepted [8,13,14,15]. Al predominantly accumulates in the root apoplast, which contains 30–90% of the total absorbed Al. It was reported that 85–90% of the total Al that is accumulated by barley roots is bound to the cell walls [16]. This reduces the movement of the water through the apoplast. Consequently, the structural properties of the cell wall change [17]. Changes in the cell wall composition in response to Al stress have been shown in detail in maize [18,19] and wheat [20,21].

The plant cell wall is mainly composed of cellulose, hemicellulose, pectins, and a small quantity of structural proteins. The pectin family, which is the most structurally complex group of polysaccharides, is a major component of the higher plant primary cell wall, comprising up to 2–10% of the primary cell wall of grasses [22]. The composition of pectin changes in response to the action of biotic and abiotic factors [22,23,24]. Pectins, especially non-esterified pectins, are thought to be the molecules to which Al binds in the apoplast [25,26,27]. The binding of Al to the pectin matrix of the cell wall makes it thick and rigid, which affects the normal expansion and elongation processes [8,9]. The binding of Al^3+^ ions to pectin depends on the negative charge, which is determined by the degree of pectin methylation [28]. It is also known that some pectin sidechains, such as arabinan and galactan, undergo modification under diverse abiotic stresses [29,30] but, to date, they have not been analyzed in response to aluminum.

Al toxicity is a major factor that seriously limits the production of barley on acidic soils [10]. Despite some evidence that Al can affect the thickness and composition of the cell wall in barley cells [17], there is still a lack of detailed knowledge on action of Al in the cell walls at the molecular level. Since the structural complexity of cell wall components is high and changes in a spatial and temporal manner, biochemical methods are not fully informative in determining the tissue- and cell-specific changes in the distribution of individual epitopes within the walls. Immunohistochemical techniques using monoclonal antibodies (mAB) enable the cell wall microstructures to be differentiated and the precise in muro localization of the polymers within complex tissues to be determined [31]. This approach can be useful in identifying the specific pectic epitopes that might be changed as a result of aluminum stress. In this study, we analyzed the presence and distribution of selected specific pectic epitopes with different degrees of methyl-esterification in the response of barley roots to aluminum stress. We discuss the obtained results in relation to the proposed involvement of pectins in regulating cell differentiation as a reaction to abiotic factors. Additionally, histological analyses were used to determine whether Al exposure leads to histological changes in barley root tips in order to elucidate their role in root elongation and expansion.

## 2. Results

### 2.1. Root Histology is Altered by Al Treatment

**Figure 1:**
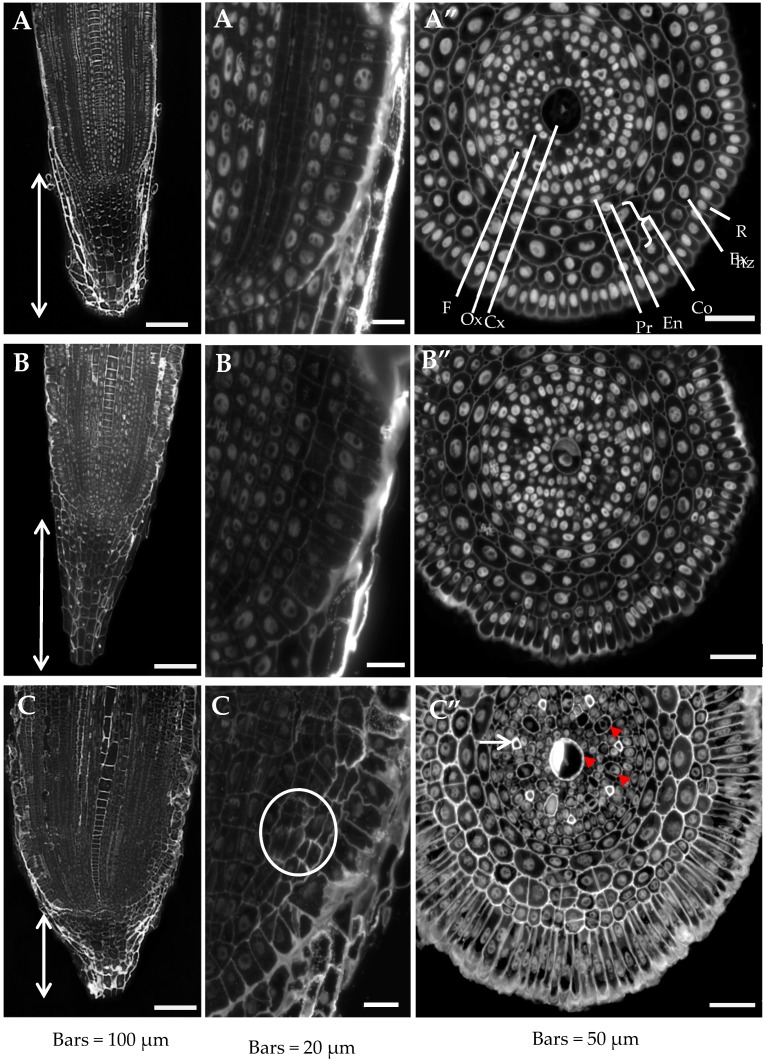
The representative longitudinal sections in meristematic zone (**A**,**A’**–**C**,**C’**) and cross sections in the differentiated zone (**A”**–**C”**) of roots of barley cv. Sebastian. Calcofluor (CF) and DAPI staining. (**A**–**A”**)—control pH 6; (**B**–**B**”)—control pH 4; (**C**–**C”**)—30 µM AlCl_3_. In A, B, and C, the root cap length is marked. (**C’**)—atypical cell divisions in exodermis and cortex are encircled. (**C”**)—red arrowheads show central and outer metaxylems with thickened walls, arrow shows xylem fiber. Rhz—rhizodermis, Ex—exodermis, En—endodermis, Co—cortex, Pr—pericycle, Cx—central xylem, Ox—outer xylem, F—fiber.

Histological analyses of the Al-treated roots in pH 4.0 and control roots grown in pH 4.0 and pH 6.0 were performed using cross sections from the differentiated zone and longitudinal sections from the meristematic zone stained simultaneously with DAPI, to distinguish the nuclei, and calcofluor (CF), which specifically detects cell walls. Differences were observed in the histology of the roots grown under different conditions (Figure 1). In the roots grown in the presence of Al, a statistically significant shortening of the root cap was observed (Figure 1A–C). The mean length of the root cap of a control root at pH 6.0 was 277 µm ± 19.5 (SD), while in the roots that had been treated with aluminum, it decreased by almost 50% and was 160 µm ± 31.7 (SD). No significant differences were observed in the lengths of the caps of the roots grown at pH 6.0 and pH 4.0 (280 ± 19.5 µm). An increase in the mean root diameter from 280 µm in control roots grown at pH 6.0 and pH 4.0 to 350 µm in the Al-treated roots was also visibly different (Figure 1A–C). This increase in the root diameter was the result of a substantial increase in the dimensions of the rhizodermal cells in the radial direction. Another reason for the change in root diameter was the increased number of cortex layers that resulted from the divisions of both the outer layer of the cortex cells and the exodermis (Figure 1C’). The mean rhizodermal cell dimension in the radial direction in the control roots at pH 6.0 was 24.7 ± 3.5 µm, while in the Al-treated roots, it increased significantly to 63.3 ± 7.5 µm. A much smaller, although statistically significant, increase in the size of the rhizodermal cells was observed for the roots grown at pH 4.0 without any Al^3+^ ions (29.8 ± 3.5 µm). An increase in the number of divisions of the cortex layers, although considerably weaker, was also observed in the roots grown at pH 4.0 (Figure 1B’,B”).

An analysis of the longitudinal sections of the roots clearly showed disturbances in cell division in the root meristematic zone, which led to an altered cortex cell arrangement (Figure 1C’, marked with a circle). Moreover, the Al-treated roots had clearly differentiated central and outer metaxylem vessels (Figure 1C” arrowheads) and xylem fibers (Figure 1C” arrow) compared to the control.

The obtained results indicate that under the influence of Al and low pH, the cell divisions in the meristematic zone are modified, the size of rhizodermal cells increases, and the differentiation and maturation of the stele is accelerated.

### 2.2. Al Treatment Affects the Distribution of the Pectic Epitopes in Barley Roots

The distribution of the LM5, LM6, LM19, and LM20 pectic epitopes (Table 1) in the roots was analyzed after 7 days of the growth of seedlings in a control solution at pH 4.0 and pH 6.0 and in seedlings treated with Al at pH 4.0. The specific pectic epitopes were used due to their different structure and possible role in Al-response.

#### 2.2.1. LM5

**Figure 2:**
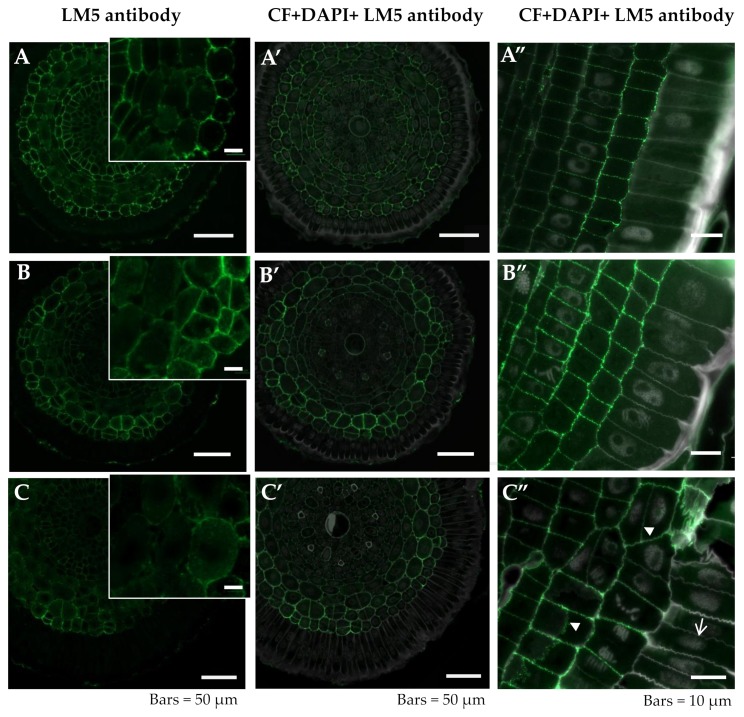
Distribution of LM5 epitope in cross sections in differentiated zone (**A**,**A’**–**C**,**C’**) and longitudinal sections in meristematic zone (**A”**–**C”**) of root tissues of barley cv. Sebastian. (**A**–**A”**)—control pH 6; (**B**–**B”**)—control pH 4; (**C**–**C”**)—30 µM AlCl_3_. (**A**–**C**) insets—higher magnification of cortex cells. Detection of LM5 epitope (**A**–**C**), Calcofluor (CF) and DAPI staining with immunolocalization of LM5 epitope (**A’**–**C’**, **A”**–**C”**) are shown. (**B”**,**C”**)—arrowheads show newly arising cell walls in exodermis and cortex with presence of LM5 epitope, arrow shows the newly formed anticlinal wall with LM5 epitope absent. Epitope distribution in representative sections are shown.

In the control roots grown at pH 6.0, the LM5 epitope was abundantly present in the exodermis, endodermis, and pericycle of the differentiation zone cells, and in the anticlinal and inner periclinal cell walls (Figure 2A,A’, Table 1). The remaining layers of the root cortex were not rich in this epitope. The central cylinder of the barley root comprised one large central and seven smaller peripheral metaxylem vessels, which were not labeled. The pectic epitope that is recognized by the LM5 antibody was not present in the rhizodermis. The distribution of the LM5 epitope in the differentiation zone in the control roots grown at pH 4.0 was similar to the control roots grown at pH 6.0 in the case of the exodermis, endodermis, and pericycle (Figure 2B,B’). In these roots, the occurrence of anticlinal and also periclinal divisions of the exodermis cells was observed. In the Al-treated roots, the LM5 epitope was present in the newly arising walls that resulted from the divisions of the exodermal and cortex cells (Figure 2C” arrowheads); however, it was not detected in the rhizodermis, even in the newly formed anticlinal walls (Figure 2C” arrow). A comparison of the LM5 distribution in the meristematic zone with its distribution in the differentiation zone showed that the cell differentiation was accompanied by a decrease in the signal (Figure 2A”,B”,C”).

#### 2.2.2. LM6

**Figure 3:**
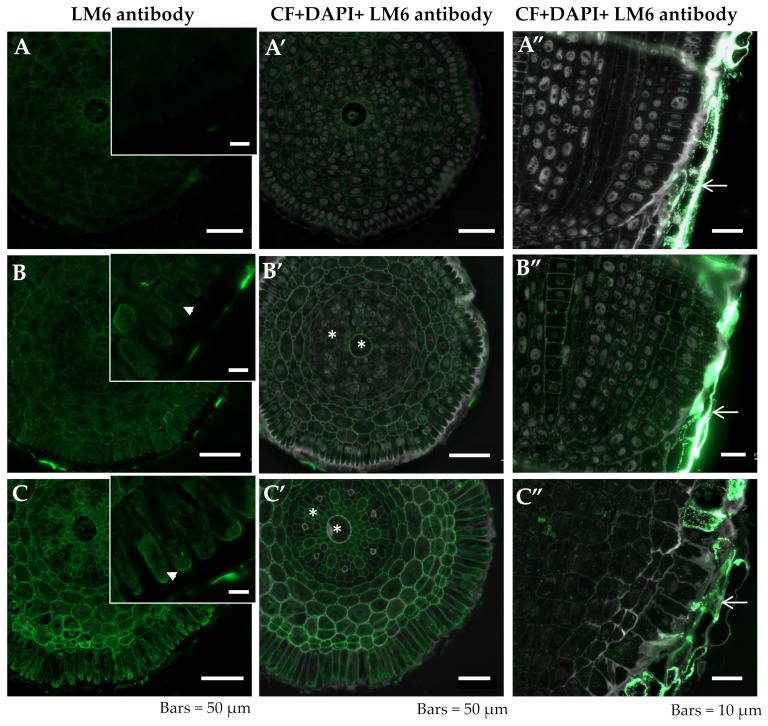
Distribution of LM6 epitope in cross sections in differentiated zone (**A**,**A’**–**C**,**C’**) and longitudinal sections in meristematic zone (**A”**–**C”**) of root tissues of barley cv. Sebastian. (**A**–**A”**)—control pH 6; (**B**–**B”**)—control pH 4; (**C**–**C”**)—30 µM AlCl_3_. (**A**–**C**) insets—higher magnification of rhizodermal cells. Detection of LM6 epitope (A–C), Calcofluor (CF) and DAPI staining with immunolocalization of LM6 epitope (**A’**–**C’**, **A”**–**C”**) are shown. (**B**,**C**)—arrowheads within insets show the presence of LM6 epitope in cytoplasmic compartments in rhizodermal cells, (**B’**,**C’**)—asterisk symbols show the xylem cells with presence of LM6 epitope, A”–C” —arrows show the remnants of root cap cells with the LM6 epitope recognized. Epitope distribution in representative sections are shown.

The peptic epitope recognized by the LM6 antibody was not detected in the differentiation zone of control roots (Figure 3A,A’, Table 1). In the differentiation zone of roots grown at low pH and the roots treated with Al, the presence of this epitope was mainly detected in cytoplasmic compartments of rhizodermal cells (Figure 3B,C arrowheads, B’,C’). However, in the central cylinder, this epitope was also present in the cell wall of the central and outer xylem (Figure 3B’,C’ asterisks). The presence of this epitope in the remnants of the root cap cells was detected for all of the experimental groups (Figure 3A”–C” arrows). A comparison of the LM6 epitope distribution in the root meristematic and differentiation zone showed that cell differentiation was accompanied by an increase in the signal (Figure 3B–B”,C–C”).

#### 2.2.3. LM19

**Figure 4:**
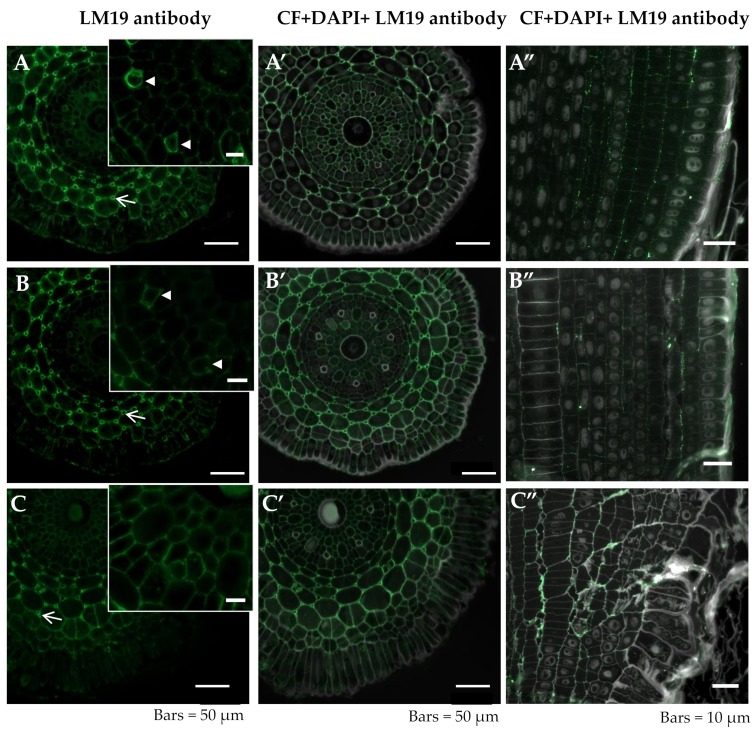
Distribution of LM19 epitope in cross sections in differentiated zone (**A**,**A’**–**C**,**C’**) and longitudinal sections in meristematic zone (**A”**–**C”**) of root tissues of barley cv. Sebastian. (**A**–**A”**)—control pH 6; (**B**–**B”**)—control pH 4; (**C**–**C”**)—30 µM AlCl_3_. (**A**–**C**) inset—higher magnification of cortex with intercellular spaces. Detection of LM19 epitope (**A**–**C**), Calcofluor (CF), and DAPI staining with immunolocalization of LM19 epitope (**A’**–**C’**,**A”**–**C”**) are shown. (**A**–**C**)—arrowheads within the insets show fibers with LM19 epitope. (**A**–**C**)—arrows show developing intercellular spaces with LM19 epitope recognized. Epitope distribution in representative sections are shown.

The LM19 epitope was primarily detected in cortex cells mainly in the developing intercellular spaces, where the regions with the maximum fluorescence were the cell junction points in all of the analyzed experimental groups of the roots in both meristematic and differentiated zones (Figure 4A–C arrows, A’–C’, Table 1). A signal was also detected within the central cylinder in the xylem and in fibers in the control roots at pH 6.0 (Figure 4A,B arrowheads). In the Al-treated roots, this epitope was present in the cortex cells, especially in the newly arising walls of the cortex cells (Figure 4) but was not detected in fibers whose development was visibly altered (Figure 4C–C’). The lowest intensity of the signal was detected in the roots grown at pH 4.0, independent of the tissue or root zone (Figure 4B–B”). In the rhizodermal cells of the control roots grown at pH 6.0, a signal was detected in the outer periclinal walls and a similar distribution was observed in the roots at pH 4.0. However, in Al-treated roots, this epitope was detected only in the cytoplasmic compartments of the rhizodermal cells, independent of the root zone (Figure 4).

#### 2.2.4. LM20

**Figure 5:**
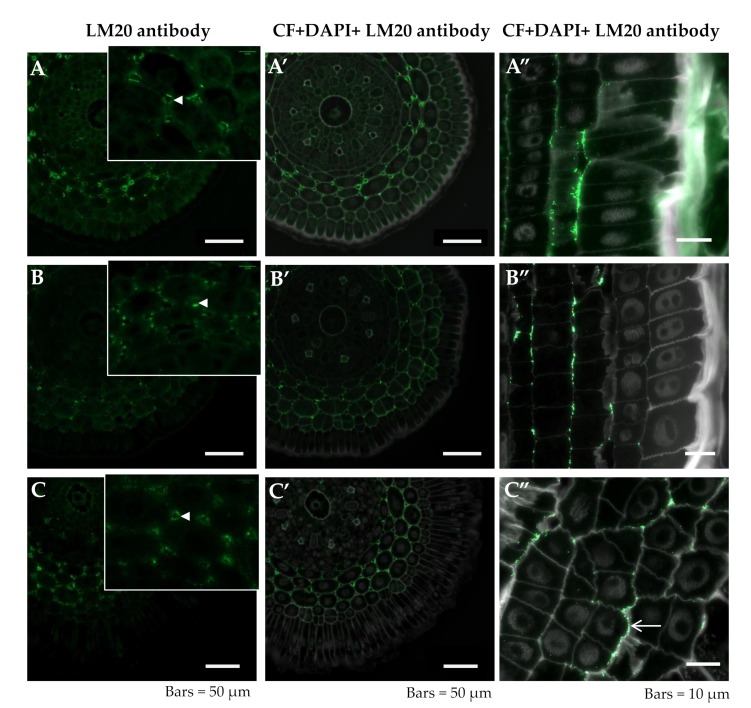
Distribution of LM20 epitope in cross sections in differentiated zone (**A**,**A’**–**C,C’**) and longitudinal sections in the meristematic zone (**A”**–**C”**) of root tissues of barley cv. Sebastian. (**A**–**A”**)—control pH 6; (**B**–**B”**)—control pH 4; (**C**–**C”**)—30 µM AlCl_3_. (**A**–**C**) insets—higher magnification of cortex cells with developing intercellular spaces. Detection of LM20 epitope (**A**–**C**), Calcofluor (CF), and DAPI staining with immunolocalization of LM20 epitope (**A’**–**C’**,**A”**–**C”**) are shown. (**A**–**C**)—arrowheads within insets show developing intercellular spaces with LM20 epitope recognized. C”—arrow shows the LM20 signal in the outer wall of cell from the complex arising from the cortex “mother” cell division. Epitope distribution in representative sections are shown.

The pectic epitope recognized by the LM20 antibody was mainly detected in the cell walls in developing intercellular spaces in the differentiation zone, independent of the treatment. The fluorescence intensity was higher in the junction points as well as in the wall facing the intercellular space (Figure 5A,A’–C,C’ arrowheads, Table 1). In the meristematic zone, this epitope was present in the periclinal walls of the cortex cells (Figure 5A”–C”). In the meristematic zone of both of the control roots, the LM20 epitope was partially distributed in the periclinal walls of the cortical cells (Figure 5 B”,C”). However, in the Al-treated roots, this epitope was only detected in the outer walls of the cell complexes arising from the cortex “mother” cell division (Figure 5 C” arrow).

### 2.3. Summary

The obtained results indicate that Al alters the growth parameters of roots and their histology, which is manifested by an increase in the root diameter, a significant increase in the diameter of the rhizodermal cells, and an acceleration of xylem differentiation and fiber maturation.

An immunohistochemical analysis revealed that Al treatment affected the cell wall chemical composition in terms of the pectin components. The distribution of all of the analyzed epitopes in the Al-treated roots was altered compared to the control. There were also differences in the distribution of pectins depending on the developmental stage of the cells that build the root body. Pectins with galactan sidechains were not characteristic of the Al-treated roots and pectins with arabinan sidechains were not detected in the control roots. Methyl-esterified homogalacturonan (HG) was not abundantly present in the barley roots, independent of the growth conditions. Based on the presented results, it can also be concluded that this epitope may be a marker of newly arising cell walls. Partially methyl-esterified HG characterized the Al-treated roots. The obtained results indicate that Al affects the cell differentiation process, thereby resulting in the inhibition of root growth under Al stress conditions.

## 3. Discussion

The primary target of Al toxicity in the root is the apex [32,33]. The first visible symptoms of Al toxicity are the inhibition of root elongation [34,35,36] and root thickening [37]. Root growth inhibition has been widely used to assess Al toxicity in numerous species [36]. A significant decrease in the root length in barley in response to Al was shown in our previous study [11]. The inhibitions of cell elongation and cell divisions are the primary mechanisms that lead to the inhibition of root growth [17]. In this work, we have shown that the thickening of the root in the differentiation zone was primarily caused by the radial increase of rhizodermal cell diameter. Although this effect was particularly noticeable after the action of Al, a slight increase in the diameter of rhizodermal cells was also visible under acidic conditions (pH 4.0) without Al treatment. The increase in rhizodermal cell diameter may somehow be correlated with the fact that it is the first layer of the root that is exposed to Al. Changes in the rhizodermis may be a manifestation of the plant defense response to various stress factors. An increase in the root diameter was also observed in barley roots exposed to Al, which was also previously reported for other plant species [12].

In the studies presented here, a statistically significant shortening of the root cap was detected in Al-treated plants. It has previously been postulated that the root cap is involved in the mechanism of Al-induced inhibition of root growth and in protecting the roots from Al toxicity [38]. Significant alterations in the mean cell volume of the root cap were detected in the primary root of *Zea mays* [39]. To the best of our knowledge, no studies to date have shown that the root cap is shortened as a result of Al. This effect may be caused by disturbances in the divisions of meristematic cells or the faster removal of cap cells that have been directed onto the programmed cell death pathway (PCD) [40]. A correlation between Al and PCD was also shown in *Nicotiana tabacum* [41], *Hordeum vulgare* [42], and *Zea mays* [43]. However, further studies are needed to determine whether the Al treatment of barley roots can accelerate PCD in root cap cells. It is commonly known that cell nuclei in the meristematic zone are the most sensitive to genotoxic factors, including Al [44,45,46]. Thus, the shortening of the root cap detected in presented studies may also be caused by a decrease in the rate of cell division in the root meristem which was previously revealed [11]. Both phenomena, the disturbance of the cell divisions and the acceleration of the PCD, can cause the alteration of the root cap that is observed under aluminum stress. It is known that the root cap participates in mucilage production, which later plays a protective role [47]. Thus, the observed decrease of the root cap length in an Al environment could be another factor that increases root decay under this stress.

Our results, which show changes in the distribution of selected pectic epitopes in barley roots treated with Al, confirm that aluminum interacts with the wall of the root cells [4]. It is widely accepted that the molecule for Al binding in the cell wall is pectin, especially the non-esterified molecule [25]. Al binds to the cell wall, electrostatically, to the negatively charged carboxyl groups of wall pectins (primarily homogalacturonan) [22], and this causes loosening and anisotropic cell expansion [15].

The function of the arabinan and galactan sidechains of pectin remains unknown. However, it was documented that the arabinan sidechains are hydrated faster than galactan sidechains [48] and, therefore, the high quantity of arabinan in the cell wall will readily result in its rehydration [23]. Moreover, the arabinans likely fulfill the function of pectic plasticizers in maintaining cell wall flexibility under abiotic stress [23]. The more abundant presence of the pectic epitopes recognized by the LM6 antibody (especially in the cytoplasmic compartments), that was detected in the presented studies, indicates that barley roots react to Al stress by synthesizing and directing the (1-5)-α-L-arabinans rhamnogalacturonan I (RG-I) sidechain to the cell wall.

The galactan sidechains of rhamnogalacturonan I (RG-I) were postulated as a component that maintains cell stiffness [49]. The decreased occurrence of this pectic epitope, recognized by the LM5 antibody, was observed in the Al-treated barley roots. The reduced cell stiffness of the Al-treated roots may explain, at least partially, the harmful effect of aluminum on plant growth. The decrease in the pectic epitope in the Al-treated plants suggests that cell expansion is limited in such roots and this is in accordance with the postulated role of the galactan-rich pectins.

In order to detect the esterified and unesterified homogalacturonans (HGs), we used the LM19 and LM20 antibodies, which are a new generation of antibodies against pectic epitopes. They are more specific then the JIM5 and JIM7 antibodies whose binding is restricted to the regions of the cell walls that line the intercellular spaces which are especially abundant at the junction between the adhered and separated primary cell walls [50]. The LM19 HG domain in pectic polysaccharides recognizes a range of HGs and prefers to bind strongly to the unesterified HG, while the LM20 HG domain in the pectic polysaccharides requires methyl esters to recognize HG and does not bind to unesterified HG [51]. Based on the studies presented here, it can be concluded that the unesterified HGs were more abundantly present in the Al-treated roots because they were detected not only in the walls that line the intercellular spaces, but also in the remaining cell walls and the cytoplasmic compartments of rhizodermal cells. Although similar results were obtained in the maize root apex, the antibodies revealed localization of the pectin in the root cortex rather than in the epidermis and stele [28]. The presence of low-esterified pectin not only in cortex cells but, also, in the rhizodermal cells of barley roots could be responsible for the response to Al and could affect sensitivity to this factor. The Al sensitivity of plants primarily depends on the degree of pectin methylation [52]. Pectin methyl esterases (PMEs), which are the enzymes that are responsible for the demethylation of pectins in the cell wall, are upregulated by Al treatment [28]. An enhancement of PME expression in the Al-sensitive maize genotype, compared to the Al-resistant one, was shown. Together with the higher pectin and Al contents in the roots of this sensitive genotype, it can be assumed that the cell wall pectins and their degree of methylation contribute to the genotypic differences in Al resistance [28]. Moreover, the results obtained in maize indicate changes in the activity of enzymes under the influence of Al. It is known that HG is exported to the cell wall in a fully methyl-esterified form and that the changes in HG esterification are the result of the methyl-esterase activity [53]. Thus, it may be concluded that the increased presence of the HG epitope by the LM19 antibody that was observed in our study after the Al treatment of barley was caused by a modified methyl-esterase activity, which is in accordance with the earlier results, thus indicating a relationship between stress factors and the activity of the cell wall enzymes [54].

The distribution of the methyl-esterified pectins recognized by the LM20 antibody in barley roots was abundant at the cell corners, where it is supposed to contribute to cell adhesion [55]. It is postulated that such a localization is correlated with the greater tension and conductivity of mechanical stresses throughout the plant tissue [56]. However, the presence of this epitope was lower (according to the signal intensity) in the Al-treated roots and in the roots grown in acidic conditions, which suggests that both of these factors may affect the mechanical properties of the root which, in turn, causes disturbances in root growth.

## 4. Materials and Methods

### 4.1. Plant Material Growth and Treatment

The roots of barley (*Hordeum vulgare*, 2n = 14) cultivar ‘Sebastian’ were used as the plant material in this study. Bioavailable aluminum Al^3+^ (30 µM) in a full Hoagland medium nutrient solution, pH 4.0, was used for the treatment. The indicated Al concentration was achieved by applying the appropriate amount of a 1 M AlCl_3_ solution. The dose of AlCl_3_ was selected based on previous experiments and the plant material was prepared for treatment as previously described [11]. Briefly, the seeds were sterilized in a 5% solution of sodium hypochlorite (Sigma, Cat. no 71696) for 15 min and rinsed in sterile water three times for 5 min. The seeds were germinated in Petri dishes covered with filter paper for 24 h at 4 °C and then for 24 h at 24 °C. The pre-germinated seeds were transferred to hydroponic cultures in 4 L containers. Two control groups were used: one with pH adjusted to 4.0 and one with pH adjusted to 6.0, in order to exclude the influence of pH on the root system of barley. Two biological replicates were set up in different containers, each containing 10 seedlings. The control and Al-treated roots were fixed for analyses after 7 days of treatment.

### 4.2. Histological Procedures

The roots were fixed in 4% formaldehyde in a phosphate-buffered saline (PBS, pH 7.3) and placed under a vacuum for 3 × 15 min (in order to remove the air from the material and to facilitate the infiltration of the fixative). After fixation, the material was washed 3 × 10 min in PBS and dehydrated in a graded ethanol series in PBS solution (30%, 50%, 70%, 90% (*v*/*v*)) each for 20 min and 99.8% ethanol twice for 20 min.

For the cross sections, roots in meristematic and differentiated zones were embedded in Steedman’s wax, which was prepared from polyethylene glycol 400 distearate and 1-hexadecanol (9:1 (*w*/*w*)) [57]. Embedding was performed at 37 °C in a graded wax/ethanol series (1:2, 1:1, 2:1 (*v*/*v*)) for 24 h each and in pure wax for another 24 h. Subsequently, the roots were embedded in pure wax and left to solidify at room temperature (RT) overnight. The root meristems were sectioned into 6 µm thick sections using a Leica RM 2145 microtome and were placed onto poly-L-lysine coated slides in a drop of water in order to stretch them. The slides were left to dry at RT overnight. Prior to the staining procedures, the sections were dewaxed and rehydrated in a graded ethanol series in a PBS solution three times for 5 min in 99.8% ethanol and once in the following dilutions for 5 min each: 90%, 70%, 50%, 30% (*v*/*v*) [24].

For the longitudinal sections, the roots were embedded in LR white resin (Polysciences). After fixation and dehydration, the material was infiltrated through an LR white resin/ethanol series of 1:2, 1:1, 2:1 (*v*/*v*) at 4 °C for 24 h each and then in pure LR white resin for another 24 h. Then, the samples were embedded in pure resin and polymerized for 48 h at 57 °C. The roots were cut into 1.5 μm-thick sections using an EM UC6 ultramicrotome (Leica Microsystems). The sections were collected onto poly-L-lysine coated glass slides.

### 4.3. Immunohistochemistry

**Figure A1:**
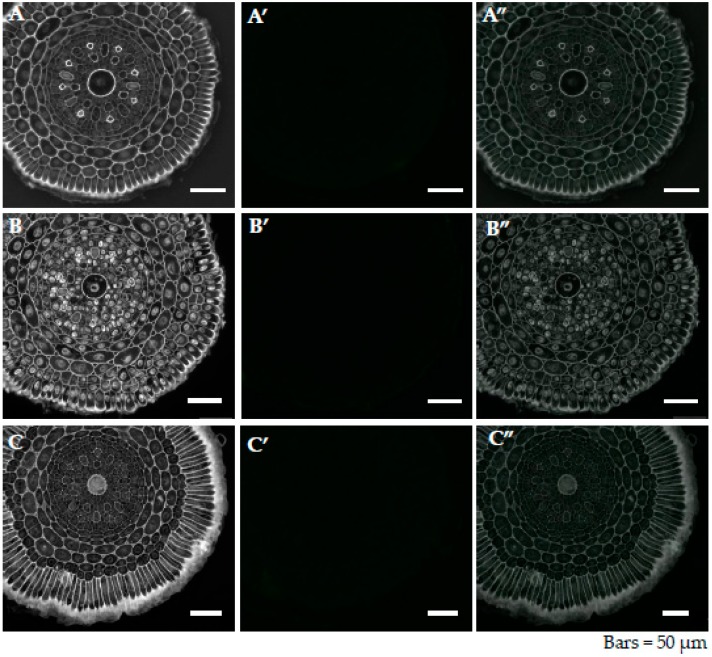
The example of the negative control for the immunolocalization of pectin epitopes in cross sections in differentiated zone of root tissues of barley cv. Sebastian. (**A**)—control pH 6; B—control pH 4; C—30 µM AlCl_3_. Calcofluor (CF) and DAPI staining (**A**–**C**), no primary antibodies in immunolocalization reaction were used (**A’**–**C’**) and merged CF and DAPI staining with negative control are shown (**A”**–**C”**). Representative sections are shown.

**Figure A2:**
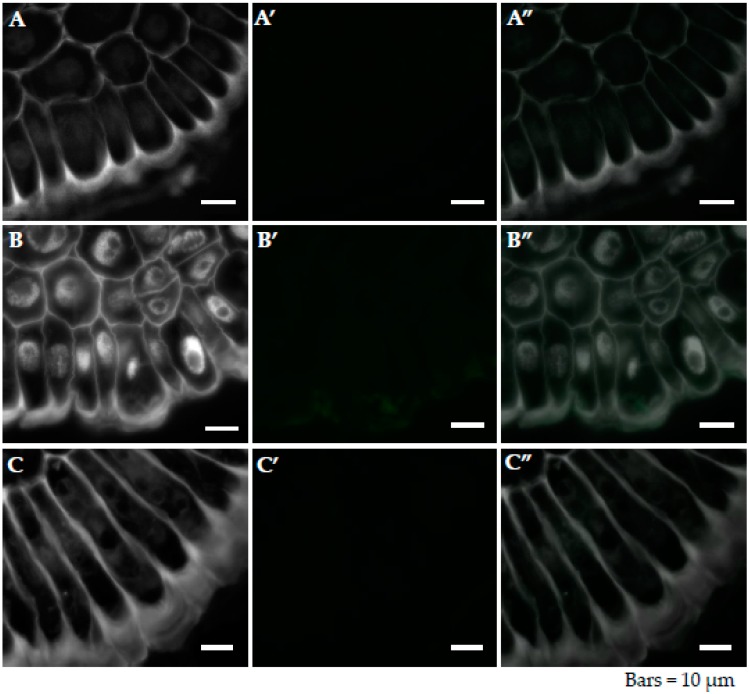
The example of the negative control for the immunolocalization of pectin epitopes in cross sections in differentiated zone of root tissues of barley cv. Sebastian. (**A**)—control pH 6; (**B**)—control pH 4; (**C**)—30 µM AlCl_3_. Calcofluor (CF) and DAPI staining (**A**–**C**), no primary antibodies in immunolocalization reaction were used (**A’**–**C’**) and merged CF and DAPI staining with negative control are shown (**A”**–**C”**). Representative sections are shown.

For the immunofluorescence labeling, all of the sections were treated with a blocking buffer consisting of 2% (*v*/*v*) fetal calf serum (FCS) and 2% (*w*/*v*) bovine serum albumin (BSA) in PBS (pH 7.3) for 30 min at RT. Next, the sections were incubated with specific primary monoclonal antibodies (Table 1), diluted 1:20 in the blocking buffer (overnight at 4 °C), rinsed with the blocking reagent three times for 10 min and incubated with the secondary antibody (Alexa Fluor 488 goat anti-rat IgG, Jackson ImmunoResearch Laboratories, West Grove, PA, USA), diluted 1:100 in the blocking buffer (1.5 h, RT). After 3 washes with the blocking reagent for 10 min and 3 washes in the PBS for 5 min, the sections were stained with 0.01% (*w*/*v*) calcofluor (Sigma-Aldrich, St. Louis, MO, USA) in PBS for 5 min, rinsed in PBS for 10 min, and then stained with DAPI (4′,6-diamidino-2-phenylindole; Invitrogen Inc.; 2 μg/mL in PBS, 5 min at RT). The slides were then rinsed with the PBS 4 × 10 min and washed with sterile distilled water 5 × 5 min. The dried slides were mounted in a Fluoromount (Sigma-Aldrich St. Louis, USA) anti-fading medium. The negative controls were performed by omitting the primary antibody step. No fluorescence signal was obtained in any control frame for any of the stained slides (Appendix A, Figure A1 and Figure A2). The presence of a greenish background on some figures was the result of autofluorescence when a longer exposure time was used. This procedure was done because of the weaker fluorescence signals from the antibodies. All of the images were taken using a Zeiss Axio Imager Z2 microscope equipped with an AxioCam Mrm monochromatic camera (Zeiss) with the corresponding software and narrow band filters for AlexaFluor 488 and DAPI.

Images were acquired for at least three root samples from two separate experiments. Histological analyses of the cross- and longitudinal-sectioned roots of the Al-treated plants and control grown at pH 4.0 and pH 6.0 were performed. Then, the distribution of the LM5, LM6, LM19, and LM20 pectic epitopes in the meristematic and differentiated root zones on the cross sections was analyzed.

To assess the size changes of the rhizodermal cells of the Al-treated and control roots grown at pH 4.0 and pH 6.0, their dimensions in the radial direction were estimated in Adobe Photoshop 4.0. The lengths of the root caps from all of the experimental groups were also estimated.

**Table 1:** List of the primary monoclonal antibodies used in the current study along with the epitopes that they recognize.

Antibody	Recognized Epitope	Reference
Pectins—rhamnogalacturonan I (RG I) and homogalacturonan (HG)		
LM5	linear tetrasaccharide in (1-4)-β-D-galactans (RG I side chain)	[58]
LM6	linear pentasaccharide in (1-5)-α-L-arabinans (RG I side chain)	[59]
LM19	unmethyl-esterified, partially methyl-esterified HG	[51]
LM20	methyl-esterified HG	[51]

### 4.4. Statistical Analysis

Statistical analysis to calculate the mean rhizodermal cell dimension in the radial direction was performed for 80 cells, analyzed in 10 cross-sectioned roots (800 in total) for each experimental group. The mean length of the root cap was estimated based on an analysis of 10 longitudinal root sections for each experimental group. The differences between the two groups were evaluated statistically using the Student’s *t*-test (*p* < 0.05). The mean ± SD is shown.

## 5. Conclusions

It can be concluded that aluminum causes tissue- and cell-specific changes in the content and localization of the analyzed pectic epitopes in barley roots, which are indicated as being the most important in the response of plants to aluminum stress. The pectic epitopes that were analyzed are involved in maintaining cell wall flexibility, stiffening the wall and firming the cells. The results indicate that specific epitopes of pectin play a role in the changes in the physical properties of the cell wall in response to stress conditions, including the pH of the growth environment and Al treatment.
